# “The importance of awareness, support and inner strength to balance everyday life” - a qualitative study about women’s experiences of a workplace health promotion program in human service organizations in Sweden

**DOI:** 10.1186/s12905-018-0704-z

**Published:** 2019-01-10

**Authors:** Madelaine Törnquist Agosti, Ingemar Andersson, Åsa Bringsén, Ann-Christin Janlöv

**Affiliations:** 1 Department of Clinical Science, Faculty of Medicine, Lund University, Malmö, Sweden; 2 Faculty of Health Science, Kristianstad University, Elmetorpsvägen 15, SE-, 291 88 Kristianstad, Sweden

**Keywords:** Woman’s health, Workplace health promotion, Work-life balance, Well-being, Human service employees, Intervention

## Abstract

**Background:**

In many European countries, women have a higher sickness absence rate than men.

Women also report higher levels of work-life conflict, which has a negative impact on women’s self-perceived health. Interventions studies on work-life balance literature are scarce. This research adds knowledge about work-life balance by examining female employees’ experiences of a newly developed intervention program, the BELE program (Balance in Everyday Life Empowerment program), aiming to enhance the work-life balance and wellbeing of female employees.

**Methods:**

All participants in the BELE program were included in the sample (*n* = 55) of the study. The methods used for gathering data were focus group interviews (*n* = 8) and semi-structured individual interviews (*n* = 8). A qualitative content analysis was used for the analyses.

**Results:**

The results showed that a majority of the participants experienced a process of change for enhancement of wellbeing and balance in their everyday life when taking part in the BELE program. In the analyses, three main themes emerged. *Reflecting and strengthening inner resource, Trying to change everyday patterns and habits and Experiencing more balance and well-being.*

**Conclusions:**

The results point out the importance of awareness and reflection on everyday life to promote work-life balance and wellbeing. The BELE program was described as a wakeup call to one’s own life and as an enhancer of empowerment processes and equality in the homes. Moreover, the results showed the need for health education in the workplace focusing on both work and private life to enhance balance and wellbeing among female employees’. The findings indicate that it is important to work at individual and group levels in work-life balance interventions and not merely at the organizational level or in the workplace setting.

**Electronic supplementary material:**

The online version of this article (10.1186/s12905-018-0704-z) contains supplementary material, which is available to authorized users.

## Background

In Europe, the sickness absence has increased rapidly and is still increasing. In many European countries, women have a higher sickness absence rate than men [1]. In Sweden, long-term sick leave with mental diagnosis has more than doubled for employees working in the public sector between 2009 and 2014 [2]. The highest long- term sick leave rate is seen among women in human service organizations. For instance, social workers and elderly care staff have the highest risk to develop long-lasting mental ill-health due to high stress and lack of recovery possibilities in the workplace [2, 3]. Furthermore, existing gender gap in the workplace and in the society effect women’s health negatively. Women report higher levels of job strain due to less decision authority compared to men and this is strongly related to depressive symptoms [4, 5]. Women also report higher levels of work-life conflicts, which have a large negative impact on women’s self-perceived health [6]. Swedish women’s experiences of work-life conflict can be related to, for instance, that they still have a greater burden for unpaid work compared to men, even though they are almost employed to the same extent as men [7, 8].

The workplace is one of the priority settings for health promotion in the twenty-first century by reaching a large number of the adult population, and as work has a significant impact on people’s health [9]. Employees’ ability to stay healthy and prosperous also increase when good terms and conditions in both work and private life exist [10]. To maintain and promote work-life balance (WLB) seems to be a key issue to effective workplace health promotion (WPH) in the organizations [11]. A positive interaction between work and private life contribute to higher overall employee well-being [12], higher job and life satisfaction [13], a greater family functioning [14] and contributes to less anxiety and depression for individuals [13].

Work-life balance has been defined in various ways and the lack of consensus make it difficult to compare research in the WLB field. The majority of the definitions of WLB in the literature has a psychological and/or sociological approach, for instance, Greenhaus and Allen [15] define WLB as the individual’s effectiveness and satisfaction in his/her work and family roles, which are consistent with life values. Moreover, WLB is also described as the accomplishment of role-related expectations that are negotiated and shared in peoples’ social contexts [16]. We believe that WLB most likely is shaped both by individual and contextual factors and it seems of importance to use a comprehensive approach in workplace health promotion with a WLB focus [17].

Although the WLB literature has increased, a comprehensive approach in workplace health promotion with a WLB focus is rare and strategies for successful support to help employees manage their work and private lives have developed slowly [18]. Furthermore, research that addresses how employees can enhance their own work–life balance is scarce [19]. Moreover, most of the WLB literature has a pathogenic approach with focus on conflict and the lack of work-life balance [20]. A majority of the WLB interventions are therefore based on studies with a conflict perspective and on organizational factors such as schedules, flexibility and work hours alone [21, 22]. These interventions and studies are all important, but they often have limited impact on employees’ personal lives [23]. A more comprehensive workplace health promotion program that includes both the organization and the individual seems favorable as it embraces individuals’ resources and strategies as well [24].

On the whole, it seems crucial to use comprehensive workplace health promotion interventions in the human service sector in order to prevent long-term sick leave and promote women’s health and well-being. This study examined female employees’ experiences of a newly developed intervention program, the Balance in Everyday Life Empowerment program (The BELE program). The BELE program is based on salutogenic research focusing on enhancing resources both in work life and private life in order to promote female employees’ WLB and wellbeing.

### Aim

The aim of the study was to explore how women working in the human service sector experienced participation in the BELE program in relation to work life balance and wellbeing.

## Methods

### Design

A qualitative approach with an inductive design was chosen since we were interested in capturing the participants’ experiences [25] of the BELE program in relation to WLB and wellbeing. The methods used for gathering data were focus group interviews (*n* = 8) and individual interviews (*n* = 8). The data collection was conducted in 2013–2014.

### Context and setting

Sweden is a welfare state, with parental, elderly and childcare benefits, subsidized through taxes. Sweden is divided in 290 municipalities. The municipalities are mainly self-governing local authorities led by politicians elected by the municipalities’ inhabitants. The municipalities are also large employers. This study was conducted together with the Social Administration (*n* = 524) and the Department of Domestic services (*n* = 107) in the municipality.

The organisations consist of a director of Social welfare services, and a director of Domestic services, department managers and heads of unit/supervisors. These leaders are all committed to following the Swedish Occupational Safety and Health Act and public health objectives. Furthermore, the human resources department in the municipality worked actively with prevention and promotion in the work environment through policies and activities. The BELE program was developed as part of the municipality’s work to promote wellbeing and health for the employees and a sustainable work life.

Many of the elderly care and personal assistant employees in the Social Administration worked part time and had shift work. The cooking staff in the Department of Domestic services mainly worked Monday to Friday but some were also working at weekends. The social workers mainly worked full-time and on a regular work schedule Monday to Friday, and if needed did overtime at weekends as well. The Social welfare services department introduced the “TimeCare” computer scheduling program in 2010, which enabled care and personal assistant employees to influence their work schedules. Social workers had flexible work hours and could to some extent self-decide start and end times/points in a workday.

### Intervention

The BELE program is a result of a research project in the municipality guided by the intervention mapping approach [26]. The aim of the program was to create possibilities for reflection, self-analysis, increased health competence and power of action to improve the balance between work and private life. Furthermore, too promote female employees’ well-being and health. The BELE program includes both a psychological and a social perspective on WLB, as WLB is most likely shaped by both individual and contextual factors [16].

The intervention included ten group meetings, two hours at a time, during a three-month period, as well as a follow-up meeting after three months (Table 1). The group meetings included brief lectures, discussions, and tasks focusing on different subjects related to well-being and WLB, and they were conducted in a supporting environment. The participants defined their own goals, which were in focus through the whole program. The group meetings were held during work time. At each meeting, the participants were given “homework” which was done during the participants’ spare time. The group leaders were the first author (MTA) and two occupational therapists working in the municipality.

**Table 1:** Sessions and content in the BELE –program

Session	Theme
1	Program introduction
2	Health/ health competence
3	Workplace health promotion/ Work-life balance
4	Occupational balance
5	Occupational pattern
6	Occupational value
7	Stress/ sleep
8	Lifestyle
9	Goal setting/ action plan
10	Action plan/ roundup
11	Reunion/ follow-up

### Procedure and participants

An invitation to participate in the research project was sent to all employees in the current departments. In the groups in 2013, all who reported their interest were invited to take part in the BELE program. In the groups in 2014, more employees were interested than the 32 available places, so lots were drawn. When taking part in the BELE program, the participants (*n* = 55) agreed to take part in a focus group interview at the end of the program and an individual interview three months after the program had finished. All participants (*n* = 55) were included in the eight focus group interviews. Eight participants were selected strategically for the individual interviews and were contacted by phone. Selection of participants for the individual interviews was based on strategic differences (age, family constellation and occupation) and reflected the composition of the workforce. The purpose of choosing participants based on differences was to explore participation in the BELE program from different perspectives, life situations and stages in life.

The participants taking part in the BELE program were women aged 23 to 64. The participants had different life situations, some were single parents with child/children, and others lived with child/children and a partner, some lived alone or with a partner. The main professions were registered nurses and assistant nurses, personal assistants, social workers and cooking staff. The women had different reasons to participate in the program, some had health problems, often caused by stress, but others just had a great interest in health education programs and/or an interest to increase their wellbeing and balance in their everyday life. A majority of the participants were healthy and working, and a minority (*n* = 2) were on part-time sick leave but still working to some extent.

### Data collection

#### Focus group interviews

Eight focus group interviews were conducted with all participants [*n* = 55] and in the same group constellations as in the program. The groups consisted of five to eight participants. Focus group methodology was chosen, as participants could listen to their colleagues’ responses and then develop their own original responses [27]. This contributed to a wider understanding of the phenomenon. Furthermore, the purpose of conducting focus group interviews was to gather data with a variety of perspectives [28] and to give all the participants an opportunity to participate and share their experiences of the program. The focus group interviews were conducted directly after the last session in the program. The focus groups consisted of participants together with one moderator (MTA), and one observer (one of the group leaders) and the interviews were held in the same conference room as the program meetings, nearby the participants’ workplaces. The focus group interviews were based on open questions as; what experience do you have of the BELE program? How has participating in the program affected you in relation to wellbeing and work-life balance? The focus group interviews lasted 1,5–2 h. All interviews were recorded digitally and transcribed verbatim.

#### Individual interviews

Eight individual interviews were conducted six months after the focus group interviews. The purpose of the individual interviews was threefold: firstly, to involve the participants in the analysis to see whether they could confirm the summary of the data collected from the focus group interviews; secondly, to contribute to a deeper understanding by offering some participants a further possibility to express their experiences of the BELE program and to elaborate on their thoughts as a result of the focus group interview; and thirdly to explore the long-term experience of the program. The individual interviews were based on an interview guide (Additional file 1) that was developed for this study and emanating from the summary of the focus group interviews. The main questions were; How did you experience your participation in the BELE program? Is there a difference compared to what your everyday life looked like before you participated in the program? Do you feel that your participation in the program has affected your well-being? A pilot interview was conducted with one of the participants in the BELE program to test the interview guide, and as no changes were made, this interview was included in the dataset. A colleague, who works at the university and who has knowledge in the research area conducted all interviews. The participants decided where the interviews should take place. The interviews lasted 1–2 h, except for one interview that was conducted by telephone. The reason for the interview being done by phone was that the participant was ill, and that interview lasted only 30 min. The interviews were recorded digitally and transcribed verbatim.

### Analysis

To answer the research question, a qualitative content analysis was used [30]. The focus groups interviews were read separately several times by the authors (MTA, A-CJ, ÅB) to provide an overall sense of the material. The data was then reflected on and discussed and brought together to a naïve understanding and a summary of the material. Meaning units from the text were then identified in relation to the aim of the study. These meaning units were condensed, abstracted and labelled with codes. Through identification of differences and similarities between the codes, further abstraction could be achieved [29].

After the focus group interviews were analyzed, the individual interviews were analyzed in the same way. In the preliminary analysis of the interviews, it become clear that the codes and categories did not differ from those in the focus group interviews about experiences of the BELE program. The individual interviews confirmed the focus group experiences but also added a deeper understanding of the women’s experiences of how the BELE program affected them over a longer period of time. Thus, all transcripts were reread and included as a whole. The texts from the individual interviews were included in the same analysis as the focus group interviews. At each step, the process of the analysis was discussed until the authors (MTA, A-CJ and ÅB) reached consensus. These three authors made the preliminary analysis, while the fourth author (IA) participated in the later steps of the analysis when the themes were developed. An example of the analysis from the text is shown in Table 2.

**Table 2:** Example of categorization procedure

Examples of Meaning units	Condensed meaning unit – description	Condensed meaning unit - interpretation	Sub-theme	Theme
I have realized by myself the way things are in reality and have a picture of it, and then things are easier and I can work on it when I know that it is somehow confirmed for me that this is the way it is and what I do and this has been very useful. (FG5)	I have realized things are in reality, which makes it easier to work on it. It has been useful and this helped me.	Awareness of everyday life creates prerequisites for change.	Reflecting and becoming aware	Reflecting and strengthening inner resources
What this program has meant for me is that something has become clear to me, what I think is important and what I think is less important as well as what I want to spend time on and what I want to un-prioritize. (FG6)	The program has clarified, what I think is important, less important, what I want to prioritize and spend my time on.	Awareness of your own values creates prerequisites for prioritizing.		
The best thing of all of this has been all the lectures we had and discussions, just the fact that we have shared experiences and knowledge because that is when I have learnt the most. (FG6)	The best thing has been the lectures and the discussions. That is when I have learnt the most.	New knowledge through exchanging experiences and lectures	Knowledge development and a change of perception	
I feel healthier, and the fact that I am now working more on myself than I did before, that I think about this and yes, I am valuable and that I have time and sort of make decisions about myself what to do and what not to do, otherwise things just roll on at work and at home. (FG4)	I am working more on myself, and I think that I am valuable and that I can make decisions about myself.	A change of perception make it easier to make healthier decisions about yourself		
“(The program) did help me to see, that if I really wanted to, I mean, how I could find balance in my life, without leaving all the decisions to others. My self-esteem is much better, like before I was so dependent on, for example my husband then, to be able to cope. (Interview 5)”	I have realized that if I really want to change something, achieve balance, I can do that without leaving all the decisions to other people.	When feeling empowered it is easier to make decisions regarding oneself.	Feeling empowered	

The researchers’ pre-understanding is part of the interpretation process [30] and was discussed in the analysis process. The first author (MTA) has been working in Social Administration departments in municipalities as a health planner and health educator to enhance workplace health. IA has many years’ experience of healthcare as a family doctor and works with health promotion research. A-CJ is a registered nurse and mental health nurse with research mainly in social services, nursing and qualitative methods. ÅB has been working as an assistance nurse and is a senior public health lecturer. Her research focuses on workplace health promotion research in healthcare settings, with a salutogenic and mixed method approach.

### Ethical considerations

The participants were informed that their participation was voluntary and that they had the right to withdraw from the study at any time. Before the interviews, the participants gave their written informed consent to participate. They were also informed that confidentiality would be preserved. This study was part of a comprehensive PhD thesis work, ethically approved by the Local Ethical Review Board of Lund Nr 2013/45.

## Results

The results showed a complex picture of how the women experienced that the BELE program promoted them to make changes in their everyday life to enhance balance and wellbeing. The women’s’ experiences of the BELE program were captured in three main themes and eight sub-themes. The first main theme was *Reflecting and strengthening inner resource* with the sub-themes *Reflecting and becoming aware*, *Knowledge development and a change of perception* and *Feeling empowered*. The second main theme was *Trying to change everyday patterns and habits* with the sub-themes *Making changes in private relations*, *Handling work life differently* and *Changing lifestyle habits.* The third main theme was *Experiencing more balance and well-being* with the sub-themes *Increasing well-being* and *A feeling of more balance in everyday life.* The results should been seen as a process where the themes interrelate. Quotes were numbered according to individual interview (IP 1–8) and focus group interview (FG 1–8).

### Reflecting and strengthening inner resources

A recurrent theme in the text was the participants’ description of how their awareness and knowledge of their own life situation increased during the program. This raised awareness and knowledge was seen as important to promote changes in everyday life. The women also talked about the BELE program content and structure as empowering.

#### Reflecting and becoming aware

The participants described that taking part in the program made them more aware of their life situation and patterns of everyday life. They highlighted that this awareness was important to their process of change and was a prerequisite for desirable changes.

*I have realized by myself the way things are in reality and have a picture of it, and then things are easier and I can work on it when I know that it is somehow confirmed for me that this is the way it is and what I do and this has been very useful. (FG 5)*

Furthermore, awareness of their own values was prerequisites to prioritizing and setting boundaries in their everyday life. Knowing their values strengthened them to stand their ground and made it possible for them to work towards their goals in life. The participants described it as a wakeup call to their own lives. The group discussions also contributed to thinking differently, to get a wider perspective and challenged their own beliefs about different subjects in life and what they “had to do”.

#### Knowledge development and a change of perception

The participants talked about the importance of knowledge in order to understand their own situation and to be able to improve their health and balance in everyday life

*The best thing of all of this has been all the lectures we had and discussions, just the fact that we have shared experiences and knowledge because that is when I have learnt the most. (FG 6)*

With the help of the program, they expressed getting a new understanding of wellbeing and health. To gain knowledge about how different health factors interact and knowledge about the determinants of health with a wider focus on wellbeing in both private life and work life were seen as contributing factors to a comprehensive picture. In the process of the program, it became easier for the participants to put words to why they felt good or bad and what was most important to their wellbeing. The women described that when they got knowledge and analyzed their everyday life and wellbeing, a change of perception usually started and a more positive and humble feeling occurred both towards themselves and others. Moreover, their trust in their own capacity to handle strain and difficult circumstances in their lives increased.

Moreover, the participants became more aware of other people’s problems with adherent solutions at the meetings. The feeling of not being alone with problems and getting the insight of being “normal” was seen as a relief, and the participants felt that they had more in common than having differences.

*- And the fact that you are not the only one, other people are also stressed and affected by their environment and whatever, and it feels so good /…/*

*- We also have very much in common, and I have discovered many things where one feels 'Oh my god, I am just the same'*

*- Yes, exactly*

*- And when you get to hear how other people handle it, you can change your ways as well, which you may not have considered before.*

*- So I think this is very good, or daring to show yourself for who you are (FG 2)*

#### Feeling empowered

With the awareness, knowledge and support within the group, the participants described themselves as being empowered. They talked about personal growth and that their self-efficacy and self-esteem was strengthened.

*“(The program) did help me to see, that if I really wanted to, I mean, how I could find balance in my life, without leaving all the decisions to others. My self-esteem is much better, like before I was so dependent on, for example my husband then, to be able to cope. (IP 5)”*

The participants also talked about how they valued themselves higher, their health and wellbeing. They described that when reflecting about what was most important in their life, it was easier to set boundaries and to prioritize themselves and their free time. Their empowerment also gave them the prerequisites to “educate” their spouse, children and others in what they learned themselves during the program. Their knowledge was shared at the dinner table and could change patterns and health behavior in the whole family. Participants also talked about how they handled their time differently, which made it possible for them to be more active with things they considered important in their life. Furthermore, a majority of the women said that they had prioritized their health more after the program and were still after six months focused on what was most important to them and their wellbeing.

### Trying to change everyday patterns and habits

Goal setting and strategies were of importance when changing a habit or a life situation. The support from the group, the group leaders, workplace and family were also considered prerequisites for achieving goals. Participants highlighted the importance of lectures, tasks, homework and discussions in their goal setting and changes. Both private life and work as well as lifestyle habits were included in the women’s goals and strategies.

#### Making changes in private relationships

Many participants said that when they started to work with themselves, they changed, and when they changed, their relationships around them also changed. This interaction was said to be crucial and mostly involved relationships and situations in private life rather than work life. The interaction could either promote a relationship or lead to the participant ending the relationship. Some of the women talked about how their relationship had become more equal as the group discussions also raised questions of equality in the family. The women expressed how much more often they had worked with domestic chores like cleaning and taking care of the children compared to their spouses.

*“It became very clear how much work I do at home and how little other people do at home, and sort of presented it in a nice way; it (the practice) was really good, and you also think yourself that it is useful, and you see that everybody needs to help out more around the house so we can all feel better (FG 2)”*

In some families, changes towards more equality in the family, due to the division of domestic chores and caring for children, improved the relationship between the parents. The women also talked about how their wellbeing was linked to the relationships with their children. It became obvious to them that their own wellbeing and health was important to the health of the rest of the family. Also, the interaction between work and home was described. When the family was harmonious and functioned well, the participants felt better, which then made them believe that they could handle their work tasks in a better way.

#### Handling work life differently

Some of the participants described how they changed the way their work tasks were organized, and their own way to handle work tasks, during and after the program. They could transform the structure of the workday to improve on doing one task at a time in order to be able to focus and be more effective. After taking part in the meeting sessions, the women said that their everyday stress did not affect them as much as it used to. The reflections and discussions about health and stress were described as coping with stress in a better way. Other participants highlighted that the workplace climate changed due to the discussions about health and stress that the participants initiated in the workplace. For instance, the importance of taking breaks during the day for recovery and to strengthen the social environment.

*“So I'm the one who has been able to make myself deal with the stress at work, to stop this unnecessary rushing around all the time; so I think about this /…/ to stop myself before I get to that level where I kind of lose all my energy or have no energy left for my free time, but have some energy left when I get home. (FG 3)”*

Some also believed that they had developed a better understanding for their co-workers and said cooperation in the workplace had improved. The participants described how their improved self-efficacy made them stand up for opinions and set boundaries at work. They talked about being more present and to reflect more about their work tasks and deadlines. Some also tried to make their work situation more comprehensible and manageable to reduce stress.

*“I guess I have opened up more at work, have gotten more courage; a problem I have had for a long time is that I have not had the courage to speak up, hold my ground and voice my opinion but instead just tagged along, thinking that things will be okay, but now: no, I don't agree, but this is what I think; this is really a big thing for me… and at the same I also feel proud then (FG 7)”*

Many of the participants also highlighted that the stress and heavy workload was to some degree depending on the organization and/or management and was out of their control to change. The program meetings were seen as an energy booster and a recovery opportunity from a hectic work situation. The tasks in the program aiming to reflecting over goals in life and how they felt about their work life made some participants question if they were in the right workplace. If not, a goal could be to look for other alternatives inside or outside the organization. Furthermore, a larger reorganization in the social welfare services department in the community was done during the BELE program, and this was often mentioned in the groups. Some of the participants felt stressed and unsecure about what was going to happen to them. Being in a group and participating in the program at this time of uncertainty was described as important to being able to reflect and cope with the situation.

#### Changing lifestyle habits

Many of the participants’ goals were to change their lifestyle habits to be more health-promoting. The participants stressed the importance of being motivated, empowered and supported by the family to be able to change their habits. During the program, some participants expressed a need for more time to unwind and recovery activities. To have time off and not always being fully booked both in work and in private life was seen as important.

*“Well, I have become more aware of what I'm doing /…/and I have been thinking that I may need to take something away, sort of thinking about what to prioritize and not rush around so much at work and in my everyday life, in my own free time, maybe take something away and have an evening off, which I have managed to do now and I think it's great (FG 4)”*

Furthermore, some women changed their everyday patterns to be able to promote good sleeping and eating habits, and the participants often started to prioritize more time for workout and exercise. This had a great deal to do with rescheduling of the private life and the partner taking more responsibility for children and domestic chores.

*“I have the same amount of time every day as everybody else, and I have sort of rescheduled it to accommodate training and time for myself, and this is really amazing (IP 6)”.*

### Experiencing more balance and well-being

All participants made some changes in their everyday life to enhance their wellbeing. After having made changes in their everyday life, the participants often experienced an increased wellbeing and some an increased work-life balance. Most participants talked about balance in everyday day and not so much of WLB.

#### Increasing well-being

A majority of the participants talked about how their wellbeing had increased during and after the program. Their goals and the changes they had completed made them gain more energy and feel better, some slept better, some exercised more, some felt happier, some had better relationships in the family, some hade reorganized their work tasks and some were less stressed, which affected their wellbeing in a positive way. The women also talked about the importance of their self-efficacy increasing, which made them feel better about themselves as well. Their wellbeing was also linked to their change of perception, to letting go of all the unnecessary musts, not taking responsibility for everyone else and prioritizing their own health and wellbeing.

*“I have also increased my self-esteem, can't really say what it is, but I feel much better inside, I can talk more and feel happier, and most of all feel that I can really handle this at work (FG 2)”*

Some participants also highlighted that even if their wellbeing had not been affected so much, they now had more tools to manage their health, wellbeing and life. The time aspect was also stressed since some changes took more than the six months the BELE program lasted. Furthermore, some women said that their change process stopped when the program ended due to less support and more resistance in the family, for instance related to domestic tasks. Rescheduling everyday life was not always easy since for some women they had to rescheduling a whole family to gain some time off for themselves.

#### A feeling of more balance in everyday life

Depending on the goals the participants had chosen to work with in the program and their life situation, the balance in their everyday life was affected more or less. For instance, changed routines at home with more equal responsibility for children and domestic chores could promote balance. Some also changed routines at work and work hours.

*“Yes, it really has (gotten better), I have made other demands and made it clear that this is not what I want, and things have changed, they have to take more responsibility, and yes, my husband has also had to take more responsibility; so things have changed a bit, so it's not just me doing everything /…/ so (the balance) has become better /…/ and I have also had a dialogue at work (IP 1).”*

The majority of the women also talked about how their new knowledge made it easier to think about balance in everyday life. Some of the women talked about balance between work and private life and others about balance in private life as well as in work life and how to prioritize. Moreover, they emphasized how important it was to think about life as a whole. If they felt good in themselves and in their family, this also affected work and the other way around. Furthermore, some participants said that it had become easier to let go of their work in their free time, to relax and to set boundaries between work and private time after the program.

## Discussion

The findings of the study indicated that a majority of the women started a process of change to enhance wellbeing and balance both in everyday life and between work and private life when taking part of the BELE program. *Reflecting and strengthening inner resources* seemed to be key components to enhancing balance in everyday life. The participants described the BELE program as a factor creating the prerequisites for awareness and knowledge through reflections and self-analyses of everyday life. Awareness is often described as the first step in the change process and the first step in self-management [26]. To use self-analyses has been shown to be effective in other studies, for instance, when returning to work after stress-related sick leave for women [31], and it seems of great importance for women to gain this knowledge as a preventive measure.

By mapping their resources, WLB and everyday life, the participants became more aware of their life situation with both possibilities and limitations. This process and knowledge made it possible to make sustainable and realistic modifications in their everyday life, which can be related to a development of a higher sense of coherence [32]. This process was described as strengthening and a feeling of empowerment often occurred. This empowerment process can be linked to health competence and self-care as key factors to enable the women to exert control over their health and to make informed choices [33, 34].

The promotion of reflection in the BELE program seems to have given prerequisites for transformative learning [35]. When the women looked at the whole system (the work, family, themselves, etc.) they could identify options for change and with critical thought step outside the system, draw on new ideas, and identify a new way of achieving change [36]. In order to step out of the box, the participants highlighted observational and participatory learning as important resources for change. In the group, they could observe how other group members handled their health and balance. Moreover, also learn their own new behaviors and get supervision from group leaders and group members in the development of new knowledge and skills for behavioral change. The group was described as an important source of support in the process of *trying to change everyday patterns and habits* and helped the women to build up courage, self-efficacy and also to test their beliefs and values. In other studies, peer group support has been shown to be effective and to increase WLB and satisfaction in both private life and work life [37] and to developing work-life coping abilities by sharing experiences [12].

The results showed a change of perception in the participants’ everyday life by focusing on what works, ones resources and a more positive approach to life. This change of perception can be related to the broaden-and-build theory of positive emotions [38]. Positive emotions appear to broaden peoples’ momentary thought action repertoires and also to build their personal resources, initiating an upward spiral towards emotional and physical wellbeing and also making it easier to cope with adversity [39]. This may explain why some of the women expressed that they felt less stress even though they described that not much around them had changed. When individuals have a positive attitude to life, it may help them to function more effectively in various roles in life and achieve a higher level of wellbeing [12]. Furthermore, the positive crossover effect [40] between family members could be seen in the results.

Hence, the participants did not always use positive emotions to achieve their goals. Instead, with the support of the group and a higher self-efficacy, they changed their everyday life by confronting a spouse or the employer. When using psychological flexibility, the participants have the potential to effectively use emotions, thoughts and behavior to achieve the best outcome in varying situations [41]. The overall results in this study confirm research that has shown that to achieve WLB and job and family satisfaction it is important that the employees believe in their own capabilities and are equipped with essential resources [42].

An important issue to raise in relation to the results of *trying to change everyday patterns and habits* and *experiencing more balance and wellbeing* is the gender inequality addressing the care giving and domestic chores. Many women in the study had to start changing the patterns of a whole family in order to find some time for self-facilitation. If the family, especially the spouse, did not support the participant, it could be difficult for her to achieve her goals in the program. The results can partly be explained by gender stereotypes, that the women have served as the primary care-giver and responsible for domestic chores which are key components of femininity, and for men being the family’s main provider is a key component of the definition of masculinity [43]. Then it is problematic that Swedish women are employed almost to the same extent as Swedish men, but they still have the larger burden for unpaid work [8]. The high work burden of Swedish women has been used as an explanation to the high sick leave rates among Swedish women compared to other European countries [44]. Hence, it is of great importance to challenging stereotypical masculinities in household work and family responsibilities for promoting women’s health [45].

### Methodological issues

In qualitative studies methodological aspects and steps must be considered and taken to help ensure that as far as possible that the findings of the study is the experiences and ideas of the informants and not the researchers [46]. Hence, the methodological aspects of credibility, dependability and transferability need to be considered for trustworthiness [29].

From a credibility perspective method triangulation was used and the individual interviews was performed by another researcher then the focus group interviews. To have an understanding of the process, progress and program, the group leaders led the focus group interviews. To minimize the risk of overemphasizing the “good” in the study, the participants, the group leaders and the researcher discussed the importance of sharing all experiences and views on the program, both positive and negative ones. The individual interviews were led by an external researcher. A disadvantage of using an external researcher can be the lack of knowledge of the program and the underlying ideas. Furthermore, to establish credibility, the interview text was read several times, analyzed separately and discussed by three researchers. Participants were also included in the analysis process by taking part of the summary of the data collected from the focus group interviews. Examples of the abstraction and interpretation process are also shown in Table 2 and citations are used throughout the presentation of the result. From a dependability perspective, the individual interviews were conducted over a short period of time to minimize the risk of inconsistency of data collection and analysis. The participants replied to the same main questions in the focus groups and in the individual interviews. The transferability is enhanced by descriptions of the context, the participants, the data collection and the analysis process, but it is the reader’s decision whether or not the findings are transferable to other context [29].

Several limitations of the study should be noted. One limitation of the study could be that the participants themselves selected to take part in the study and the BELE program hoping to improve their wellbeing and balance in everyday life. However, in health promotion interventions it seems to be particularly beneficial for self-selected samples [47]. Another limitation could be the focus on the participants’ experiences of the BELE program in relation to WLB and wellbeing, which leave out other experiences of the program. Moreover, as this was a qualitative study, the aim was not to generalize but to gain a deeper understanding about participants’ experiences of the BELE-program in relation to wellbeing and WLB.

### Implications

The findings of this study have several practical implications for organizations and individuals. The findings show that apart from working with structural changes such as schedules and parental leave, the organizations should also offer individual solutions to enhance WLB and wellbeing. The framework of the BELE program create possibilities for reflection, self-analysis, increased health competence and power of action in the different domains of work, private life and the individual, which seems to enhance WLB and wellbeing. It also seems favorable to use a salutogenic approach in health promotion work in the workplace focusing on balance in everyday life. The salutogenic perspective could complement the more often used pathogenic approach in the HR departments to enhance resources and create upward spirals towards WLB and wellbeing among employees.

## Conclusions

This study’s results add to the existing knowledge often based on a conflict approach with an organizational focus to WLB. The findings of the study clearly indicate the importance of a holistic perspective, including the individual, private life and workplace aspects when analyzing everyday life and improving the health related knowledge and awareness among women. To give women the opportunity to reflect on and strengthen inner resources seems to be a prerequisite for changing everyday patterns and habits to achieve a more health-promoting life. The content and structure of the BELE program also raised questions of equality, which is a key issue for health promotion work in all areas of the society. To work for higher visibility of women’s life situations in all areas of society is of great importance to improve gender equality and women’s health. Furthermore, to be able to change women’s life situations, it seems necessary to develop methods of high quality in health-promoting work. The development of the BELE program was an attempt to contribute with a method for enhancement of women’s WLB and wellbeing. Hence, the BELE program needs to be further investigated and evaluated in relation to women’s health and sick leave rates

## Additional file

Additional file 1: Interview guide. (PDF 69 kb)

## Abbreviations

BELE program: Balance in Everyday life Empowerment program
WLB: Work-life Balance
WPH: Work Place Health promotion
