# Supplementary material for: “The importance of awareness, support and inner strength to balance everyday life” - a qualitative study about women’s experiences of a workplace health promotion program in human service organizations in Sweden
Source: BMC Womens Health. 2019 Jan 10;19:7. doi: 10.1186/s12905-018-0704-z (PMC6327387; doi:10.1186/s12905-018-0704-z)
Supplement: Supplementary file 1 — Interview guide. (PDF 69 kb) [file 12905_2018_704_MOESM1_ESM.pdf]

Interview Guide

- How did you experience your participation in the BELE program?
- Please tell me what your everyday life looks like at present, six months after the program was completed.
- Is there a difference compared to what your everyday life looked like before you participated in the program? If there is a difference, please describe how it is different? Positive, negative?
- Do you feel that your participation in the program has affected the balance between your work and your private life? If it has, please describe how it has been affected.
- Do you feel that your participation in the program has affected your well-being? If it has, please describe how it has been affected.

In the preliminary focus group analysis, we found that the participants highlighted some areas where changes had occurred during and/or after the program. Do you feel that there were changes for you in these areas? If yes, in what way?

- **Your work**

Do you feel that there are some aspects in your work domain that have changed? If yes, which ones and how have they changed?

- **Family/Social Network**

Are there some aspects within the family/social network that you feel have changed? If yes, which ones and how have they changed?

- **Personally**

Are there some of your personal aspects that you feel have changed? If yes, which ones and how have they changed?

- Are there other areas you would like to highlight as you feel they have been affected by the program? Positively? Negatively?
- Is there something that you feel in retrospect should have been included in the program? Is there something you would like to change?
- Is there something else you would like to tell us about the program and how you experienced it?
